# SNAI2-Induced CircMTO1 Promotes Cell Proliferation and Inhibits Apoptosis Through the miR-320b/MCL1 Axis in Human Granulosa-Like Tumor Cells

**DOI:** 10.3389/fgene.2021.689916

**Published:** 2021-08-03

**Authors:** Jie Duan, Hongning Cai, Yanming Huang, Liangyan Shi

**Affiliations:** ^1^Department of Gynecology, Maternal and Child Health Hospital of Hubei Province, Wuhan, China; ^2^Department of Gynecology, Women and Children’s Hospital of Hubei Province, Wuhan, China; ^3^Department of Gynecology II, Maternal and Child Health Hospital of Hubei Province, Wuhan, China; ^4^Department of Gynecology II, Women and Children’s Hospital of Hubei Province, Wuhan, China

**Keywords:** circular RNA, circMTO1, miR-320b, MCL1, SNAI2, polycystic ovaries syndrome

## Abstract

Polycystic ovary syndrome (PCOS), one of the most common types of endocrine diseases, is characterized by a high prevalence among women of reproductive-age. However, its pathogenesis and molecular mechanisms remain unclear. CircMTO1 has been reported to participate in numerous biological processes, but, its role in PCOS progression remains unknown. In the current study, we elucidated the expression and circRNA characterization of circMTO1 in human granulosa-like tumor cells. We found that circMTO1 knockdown promoted human granulosa-like tumor cell proliferation and inhibited its apoptosis rate. Next, we explored the underlying molecular mechanisms by using a series of experiments. Our results revealed the effect of the novel circMTO1/miR-320b/MCL1 axis in human granulosa-like tumor cells. Furthermore, we found that the expression of circMTO1 was induced by Snail family transcriptional repressor 2 (SNAI2) in human granulosa-like tumor cells. Our results may provide potential targets for PCOS research and a novel direction for the diagnosis and treatment of PCOS.

## Introduction

Polycystic ovary syndrome (PCOS),one of the most common types of endocrine diseases, occurs in about 5–10% of women of reproductive age worldwide (Azziz et al., 2004). Multiple risk factors, such as hyperinsulinemia, polycystic ovaries, and dysfunction of the ovulatory cycle (Azziz et al., 2006; Diamanti-Kandarakis and Dunaif, 2012) lead to its high prevalence and considerable burden for clinical management. However, the pathogenesis and molecular mechanisms of PCOS remain poorly understood. Therefore, finding new potential targets for understanding the progression of PCOS is urgently needed.

Previous studies have demonstrated that granulosa cells (GCs) may play an essential role in PCOS development. In comparison with GCs from women with normal ovulatory cycles, GCs from PCOS patients have lower proliferation and apoptosis ability than GCs from women with normal ovulatory cycles (Das et al., 2008). Human granulosa-like tumor cells (KGN and SVOG) have similar pathological features to GCs, and could be well-cultured (Nishi et al., 2001); therefore, KGN and SVOG cells have been widely used in PCOS research (Zhang et al., 2010; Han et al., 2018; Li M. et al., 2019; Chen et al., 2020). Indeed, human granulosa-like tumor cells represent a promising research direction for PCOS therapeutic targets.

With the technological innovations in high-throughput sequencing (RNA-seq) over the past two decades, the structure and biological functions of circular RNAs (circRNAs) have been intensely studied (Patop et al., 2019). CircRNA is a covalently closed RNA without polyadenylation (Jeck and Sharpless, 2014) and is abundantly expressed and conserved in eukaryotic organisms (Jeck et al., 2013). Mechanistically, circRNAs are mainly considered as post-transcriptional regulators that act as sponges for microRNAs (miRNAs) and affect the binding capability of messenger RNAs (mRNAs) to downstream factors (Hansen et al., 2013; Memczak et al., 2013). Circular RNAs interact with proteins and even translate proteins (Hentze and Preiss, 2013; Legnini et al., 2017; Yang et al., 2017). Biologically, the essential roles of circRNAs in multiple cellular processes have been widely reported, including proliferation, apoptosis, migration, invasion, and angiogenesis (Bian et al., 2018; Liu et al., 2018; Ouyang et al., 2019; Song et al., 2019; Zhou et al., 2019).

CircRNA expression profiles have been studied in detail in PCOS (Che et al., 2019; Wang et al., 2019; Zhang C. et al., 2019). Lu et al. (2020) found that CiRS-126 acts as a sponge for miR-21 to inhibit ovarian granulosa cell viability. Deng et al. (2020) reported that circPUM1 regulates PCOS development by interacting with miR-760. These studies indicate the crucial role of circRNAs in PCOS progression. CircRNA mitochondrial translation optimization 1 (MTO1 homolog; hsa_circRNA_0007874/hsa_circRNA_104135) has been found to participate in the progression of multiple diseases, including hepatocellular carcinoma, cervical cancer, rectal cancer, and acute kidney injury (Han et al., 2017; Chen et al., 2019; Shi et al., 2020). Several studies have reported the modulation of circMTO1 of cell proliferation behavior (Ge et al., 2018; Zhang X. et al., 2019; Wang N. et al., 2020). However, the involvement of circMTO1 in the cellular progression of human granulosa-like tumor cells remains unclear.

In this study, we aimed to elucidate the function of circMTO1 in human granulosa-like tumor cells. Our study revealed the expression and circRNA characterization of circMTO1 in human granulosa-like tumor cells. We performed CCK8 and flow cytometry to elucidate the cellular effects of circMTO1 on human granulosa-like tumor cells. Further, we explored the underlying molecular mechanisms and subsequently demonstrated a novel snail family transcriptional repressor 2 (SNAI2)/circMTO1/miR-320b/MCL1 axis in PCOS cellular progression.

## Materials and Methods

### Cell Culture and Transfection

Human granulosa-like tumor cell lines (KGN and SVOG cells) and 293T cells were purchased from the Cell Bank of the Chinese Academy of Sciences (Shanghai, China). DMEM high glucose (HyClone, Logan, UT, United States) supplemented with 10% FBS (BI, Israel) was used to culture cells in an environment containing 5% CO_2_ at 37°C. All lentiviruses, siRNAs, and plasmids were synthesized by GeneChem (Shanghai, China). Lipofectamine 3,000 (Invitrogen) was used to perform all transfections according to the manufacturer’s instructions.

### RNA Isolation and Quantitation and RNase R Treatment

We used TRIzol reagent (Invitrogen) was used to harvest RNA from the cells. A superscript RT Kit (TOYOBO) was used to reverse transcribe cDNA following the manufacturer’s protocol. For miRNA detection, a TaqMan MicroRNA Assay (Applied Biosystems, United States) using a specific TaqMan miRNA probes hsa-miR-320b (ID 002844) (Applied Biosystems, United States) was applied following the manufacturer’s protocols. RNA quality was assessed using a NanoDrop 2,000 spectrophotometer (Thermo Fisher Scientific, United States). GAPDH and U6 were used as the internal controls. RNA levels were detected using a 7,500 Fast PCR instrument (Applied Biosystems, United States). RNA expression fold change level was presented by the 2^–^^Δ^^Δ^^Ct^ method. For RNase R treatment, 3 U/mg of RNase R (Epicenter, United States) was used to digest 1 μg of RNA for 30 min at 37°C, following the manufacturer’s instructions. The stability of mMTO1 and circMTO1 was assessed by RT-PCR assay. Target values were assessed using the 2-ΔΔCT method. The primer sequence information was as follows: circMTO1; 5′-GAGCTGTAGAAGATCTTATTC-3′(F), 5′-CACAGGCCATCCAAGGCATC-3′(R), miR- 320b; RT primer: 5′GTCGTATCCAGTGCAGGGTCCGAGGTATTCGCACTGGA TACGACTTTTCGAC 3′, 5′ TCCGAAACGGGAGAGTTGG 3′(F), 5′ GTGCAGGGTCCGAGGT 3′ (R); MCL1; 5′-GCTGC ATCGAACCATTAGCA-3′(F), 5′-ATGCCAAACCAGCTCCT ACT-3′(R), SNAI2; 5′-GTATCTCTATGAGAGTTACTCCATGC CTG-3′ (F), 5′-TTACATCAGAATGGGTCTGCAGATGAGC-3′ (R); GAPDH; 5′ GCACCGTCAAGGCTGAGAAC 3′(F), 5′ TGGTGAAGACGCCAGTGGA 3′(R), U6; 5′ TCCGA TCGTGAAGCGTTC 3′(F), 5′ GTGCAGGGTCCGAGGT 3′(R).

### Western Blot

Total proteins were isolated using radioimmunoprecipitation assay buffer (Thermo Scientific). Protein quantification was performed using a BCA protein concentration assay kit (Solarbio). Proteins were separated by SDS-PAGE on a 10% gel and transferred onto polyvinylidene fluoride membranes (Millipore, United States). Subsequently, the membranes were subjected to 5% non-fat milk (Sangon, China) for 45 min. The membranes were then incubated with primary antibodies for 12 h at 4°C. The membranes were washed 3 times with TBS-T and then incubated with secondary antibodies for 1.5 h at 23°C. The experimental results were visualized using an image analysis system (WD-9413B, Liuyi, Beijing, China). The antibodies used in this study were as follows: MCL1 (1:1,000, CST, 94296S), GAPDH (1:1,000, CST, 5174S).

### RNA Fluorescence *in situ* Hybridization (FISH) Assay

A fluorescent *in situ* hybridization (FISH) kit (RiboBio, China) was used to conduct our RNA FISH experiment following the manufacturer’s protocol. The probes used in this study were purchased from GeneChem (Shanghai, China). The experimental results were visualized using confocal microscopy.

### Cell Viability Detection

Cell Counting kit-8 (CCK8, Dojindo, Japan) was used to measure cell viability following the manufacturer’s protocol. A 96-well plate was used to seed the cells (2 × 10^4^ cells per well). 2 days later, we added 10 μl CCK-8 solution was added to the culture cells for 120 min. The OD values of the cells were measured at a wavelength of 450 nm.

### Cell Apoptosis Measurement

The apoptosis rate of the cells was assessed using flow cytometry (BD Biosciences, United States). First, the cells were collected and resuspended in a binding buffer. Second, cells were cultured with 0.25 mg/ml Annexin V-FITC (Dojindo, Japan) and 10 mg/ml propidium iodide (Dojindo, Japan) in a dark environment at room temperature for 30 min. Finally, the treated cells were subjected to flow cytometry for apoptosis analysis.

### Biotinylated RNA Pull-Down

Biotinylated RNA probes were obtained from GenePharma (Shanghai, China). After transfection for 2 days, the cells were washed with PBS and cultured on ice for 10 min. Total RNA was isolated from the cells pre-transfected with biotinylated RNA. RNA was purified by centrifugation, and 100 ml purified RNA was collected as input. We added M-280 streptavidin magnetic beads (Sigma) to the cell lysates. Trizol was used to purify the bound RNA for analysis.

### RNA Immunoprecipitation

We used a Magna RIP^TM^ RNA-Binding Protein Immunoprecipitation Kit (Millipore, Bedford, MA, United States) for the RNA immunoprecipitation assay, following the manufacturer’s instructions. AGO2 and IgG antibodies were used. We then conducted a qRT-PCR experiment to analyze the bound RNA.

### Dual-Luciferase Reporter Gene Assay

The pmirGLO plasmids harboring the sequences of circMTO1 WT/MUT and MCL1 WT/MUT were synthesized and commercially procured from GenePharma (Shanghai, China). All the transfection processes were performed using Lipofectamine 3,000 (Invitrogen). After transfection, the luciferase activity was measured and recorded.

### Statistical Analysis

We used IBM SPSS Statistics for Windows, Version 21.0 (IBM Corp., Armonk, NY) to analyze data from the experiment results. All data are presented as the mean ± standard deviation (SD). For the measurement of data from two groups, we used Student’s *t*-test, and for data from three or more groups, we employed a one- way analysis of variance. All experiments were repeated at least 3 times unless otherwise stated. Differences were considered statistically significant at *P* < 0.05.

## Results

### Characterization and Biological Behavior of CircMTO1 in Human Granulosa-Like Tumor Cells

**FIGURE 1:**
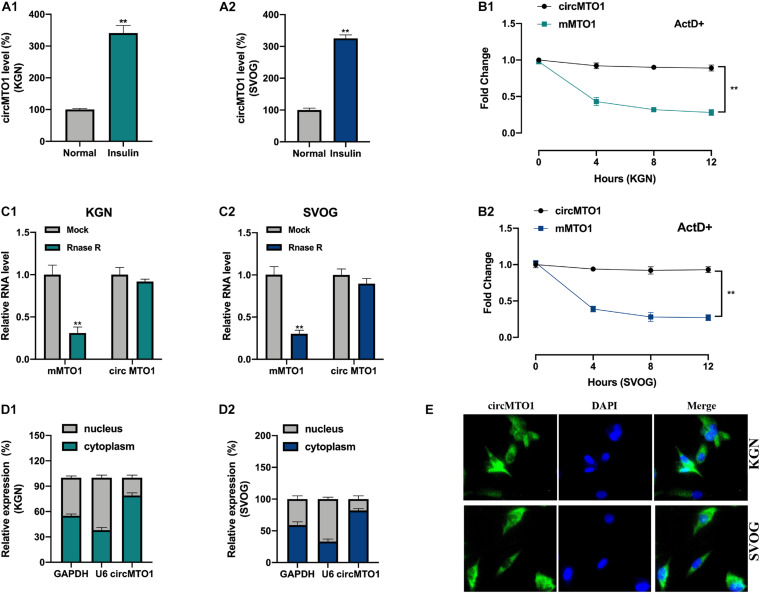
Characterization of circMTO1 in Human granulosa-like tumor cells. **(A1,A2)** Relative circMTO1 expression in insulin-treated Human granulosa-like tumor cells was detected by qRT-PCR. **(B)** Relative expression level of circMTO1 and liner MTO1 (mMTO1) in KGN **(B1)** and SVOG **(B2)** cells after treated with ActD were evaluated by qRT-PCR. **(C)** Relative expression of circMTO1 and mMTO1 in KGN **(C1)** and SVOG **(C2)** after treatment with RNase R were assessed by qRT-PCR. **(D)** The distribution of circMTO1 in KGN **(D1)** and SVOG **(D2)** cells was measured using qRT-PCR. **(E)** The location of circMTO1 in Human granulosa-like tumor cells was detected using the FISH assay. All assays were repeated 3 times, *P< 0.01.

Initially, we assessed the expression levels of circMTO1 in human granulosa-like tumor cells after insulin treatment. As shown in Figures 1A1,A2, the levels of circMTO1 in KGN and SVOG cells were upregulated upon insulin treatment. Next, we verified circRNA characterization of circMTO1 in human granulosa-like tumor cells. Upon ActD treatment, MTO1 mRNA was obviously degraded, but not circMTO1 (Figures 1B1,B2). Our results also suggested that the level of MTO1 mRNA, but not its circular form (circRNA), in human granulosa-like tumor cells was significantly decreased by RNase R treatment (Figures 1C1,C2). We found that circMTO1 was mainly located in the cytoplasm of human granulosa-like tumor cells (Figures 1D1,D2,E).

**FIGURE 2:**
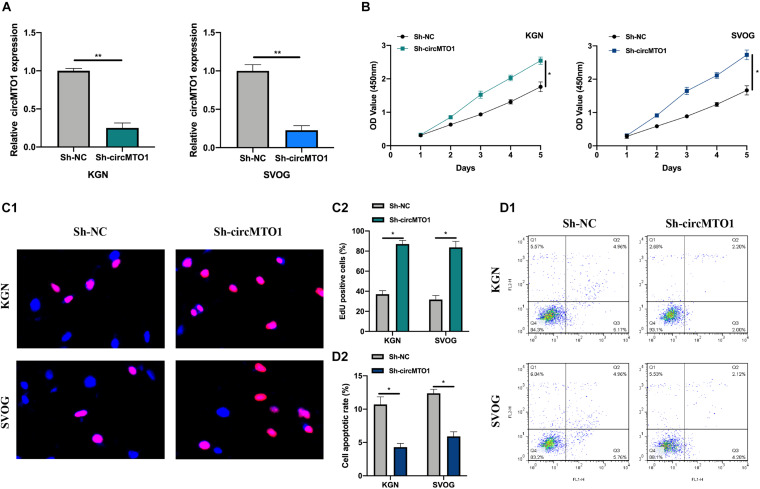
Biological behavior of circMTO1 in Human granulosa-like tumor cells. **(A)** The statistical circMTO1 knockdown cell models were constructed, expression level of circMTO1 in indicated cells was measured by qRT-PCR. **(B)** The proliferation level of circMTO1 knockdown cells was assessed by CCK-8 assay. **(C)** EdU assay was conducted to evaluate the effect of downregulated circMTO1 on the proliferation abilities of human granulosa-like tumor cells **(C1)**; statistical analysis was showed **(C2)**. **(D)** Flow cytometry experiment was conducted to measure cell apoptotic rate of circMTO1 knockdown human granulosa-like tumor cells **(D1)**, statistical results were analyzed **(D2)**. All experiments were conducted 3 times. *P< 0.05, **P< 0.01.

Subsequently, we evaluated the biological function of circMTO1 in human granulosa-like tumor cells. CircMTO1 knockdown cell models were constructed (Figure 2A). Using CCK-8 and EdU assays, we verified that circMTO1 knockdown promoted PCOS cell proliferation (Figures 2B,C1,C2). The flow cytometry experiment results indicated that circMTO1 knockdown inhibited the apoptotic rate of human granulosa-like tumor cells (Figures 2D1,D2). These results suggest that circMTO1 is involved in the cellular processes of human granulosa-like tumor cells.

### CircMTO1 as an Efficient Molecular Sponge for miR-320b

**FIGURE 3:**
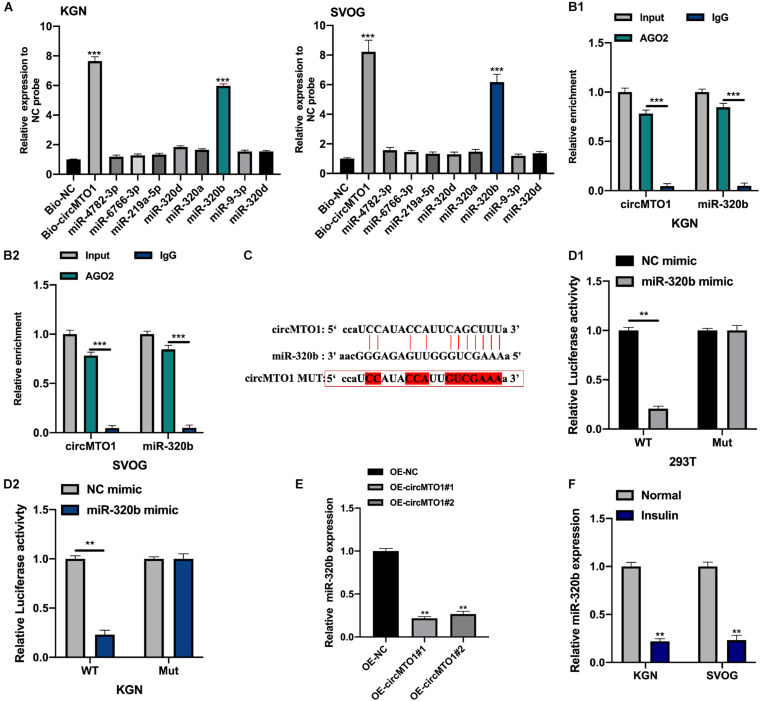
CircMTO1 as an efficient molecular sponge for miR-320b. **(A)** Relative expression of putative miRNAs was measured by qRT-PCR in human granulosa-like tumor cells after biotinylated RNA pull-down. **(B)** Relative enrichment of circMTO1 and miR-320b in anti-IgG and anti-AGO2 incubated KGN **(B1)**, and SVOG **(B2)** cells were assessed by qRT-PCR. **(C)** The binding sites between circMTO1 and miR-320b were presented. **(D)** Relative luciferase activities in 293T **(D1)** and KGN **(D2)** co-transfected with circMTO1 WT or MuT and miR-320b mimics were measured. **(E)** Relative expression of miR-320b in OE-NC and OE-circMTO1 transfected Human granulosa-like tumor cells. **(F)** Relative expression of miR-320b in insulin treated Human granulosa-like tumor cells. All experiments were performed 3 times, **P< 0.01, ***P< 0.001.

We used the Miranda dataset^1^ to predict the downstream targets of circMTO1 (CLIP-seq data: strict stringency ≥ 5). Further, we conducted biotinylated RNA pull-down and qRT-PCR assays to measure the expression of putative miRNAs in biotinylated probes. MiR-320b was found to be highly enriched in Bio-circMTO1 probes in human granulosa-like tumor cells (Figure 3A). Subsequently, we found that circMTO1 and miR-320b were abundantly enriched in anti-AGO2 purified complexes compared with anti-IgG (Figures 3B1,B2). The predicted binding sites are shown in Figure 3C. The luciferase activities in miR-320b mimic and vectors containing circMTO1 WT sequence co-transfected cells were significantly decreased (Figures 3D1,D2). Furthermore, circMTO1 suppressed miR-320b expression in human granulosa-like tumor cells (Figure 3E). MiR-320b expression in human granulosa-like tumor cells decreased after insulin treatment (Figure 3F).

### MiR-320b Directly Targeting MCL1

**FIGURE 4:**
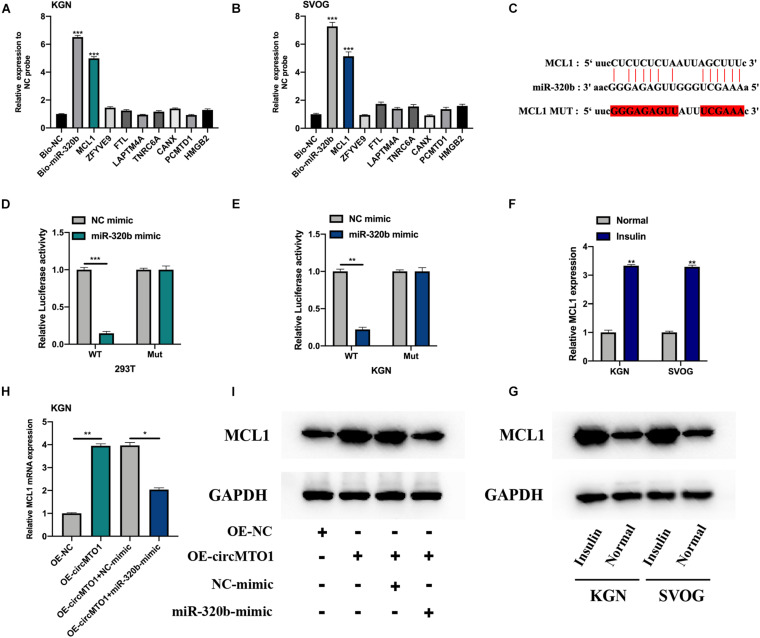
MiR-320b directly targeting MCL1. **(A,B)** Relative expression of putative mRNA targets for miR-320b in biotinylated miR-320b (Bio-miR-320b) and its normal control (Bio-NC) probes infected KGN **(A)**, and SVOG **(B)** cells were evaluated by qRT-PCR. **(C)** The binding sites between miR-320b and MCL1 were showed. **(D,E)** Relative luciferase activities in 293T **(D)** and KGN **(E)** co-transfected with circMTO1 WT or MuT and miR-320b mimics were assessed. **(F,G)** Expression level of MCL1 in insulin-treated human granulosa-like tumor cells was measured by qRT-PCR **(F)** and western blot assays **(G)**. **(H,I)** Expression of MCL1 in OE-NC, OE-circMTO1, OE-circMTO1 + NC-mimic, and OE-circMTO1 + miR-320b-mimic transfected Human granulosa-like tumor cells were measured by qRT-PCR **(H)** and western blot **(I)**. All experiments were applied 3 times, *P< 0.05, **P< 0.01, ***P< 0.001.

We investigated the mRNA targets of miR-320b using the starBase dataset^2^. We selected 8 potential targets of miR-320b for biotinylated RNA pull-down assays (data were supported by the maximum number of Ago CLIP-seq). MCL1 was found to be highly enriched in bio-miR-320b probes in human granulosa-like tumor cells (Figures 4A,B). The predicted binding sites between miR-320b and MCL1 are shown in Figure 4C. Luciferase reporter assays confirmed the association between miR-320b and MCL1 in 293T and KGN cells (Figures 4D,E). MCL1 mRNA and protein expression was found to be increased under insulin treatment in human granulosa-like tumor cells (Figures 4F,G). CircMTO1 promoted MCL1 expression in human granulosa-like tumor cells and was reversed by the miR-320b mimic (Figures 4H,I).

### CircMTO1 Regulation of Human Granulosa-Like Tumor Cell Behaviors via miR-320b/MCL1 Axis

**FIGURE 5:**
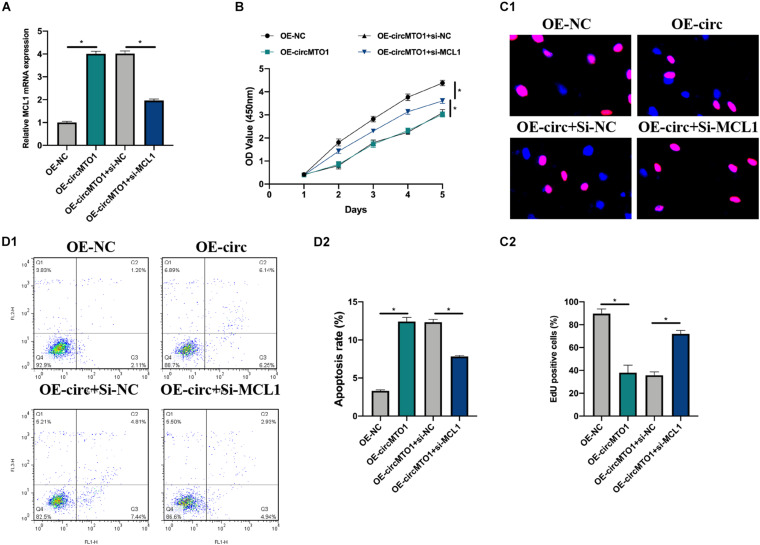
CircMTO1 regulation of human granulosa-like tumor cell behaviors via miR-320b/MCL1 axis. Cell models were generated by infecting OE-NC, OE-circMTO1, OE-circMTO1 + si-NC, OE-circMTO1 + si-MCL1 into KGN cells. **(A)** Relative expression of MCL1 in OE-NC, OE-circMTO1, OE-circMTO1 + si-NC, and OE-circMTO1 + si-MCL1 transfected KGN cells were assessed by qRT-PCR. **(B)** The proliferation of KGN cells under indicated transfections was measured by CCK-8. **(C)** EdU assay was used to evaluate the proliferation level of indicated KGN cells **(C1)**, statistical results were analyzed **(C2)**. **(D)** Cell apoptosis of GCs upon different transfections were detected by flow cytometry **(D1)**, comparative statistics were shown **(D2)**. All assays were performed 3 times, *P< 0.05.

Cell models were constructed by transfecting OE-NC, OE-circMTO1, OE-circMTO1 + si-NC, and OE-circMTO1 + si-MCL1 into human granulosa-like tumor cells to elucidate the role of the circMTO1/miR-320b/MCL1 axis. Next, we then evaluated the transfection conditions (Figure 5A). The promotive effects of OE-circMTO1 on cell proliferation were rescued by si-MCL1 (Figures 5B,C1,C2). Moreover, si-MCL1 reversed the inhibitory effect of OE-circMTO1 on cell apoptosis (Figures 5D1,D2). The results showed that circMTO1 facilitated human granulosa-like tumor cell behavior by regulating MCL1 expression via miR-320b.

### Induction of CircMTO1 Expression in Human Granulosa-Like Tumor Cells Is Induced by SNAI2

The aforementioned results revealed the novel circMTO1/miR-320b/MCL1 axis in the cellular process of human granulosa-like tumor cells. However, the upstream regulator of circMTO1 requires further investigation. By utilizing the databases of the National Center for Biotechnology Information (NCBI),^3^ Genomics Institute at the University of California, Santa Cruz (UCSC),^4^ and JASPAR,^5^ we determined that SNAI2 is a promising putative transcription factor of MTO1.

**FIGURE 6:**
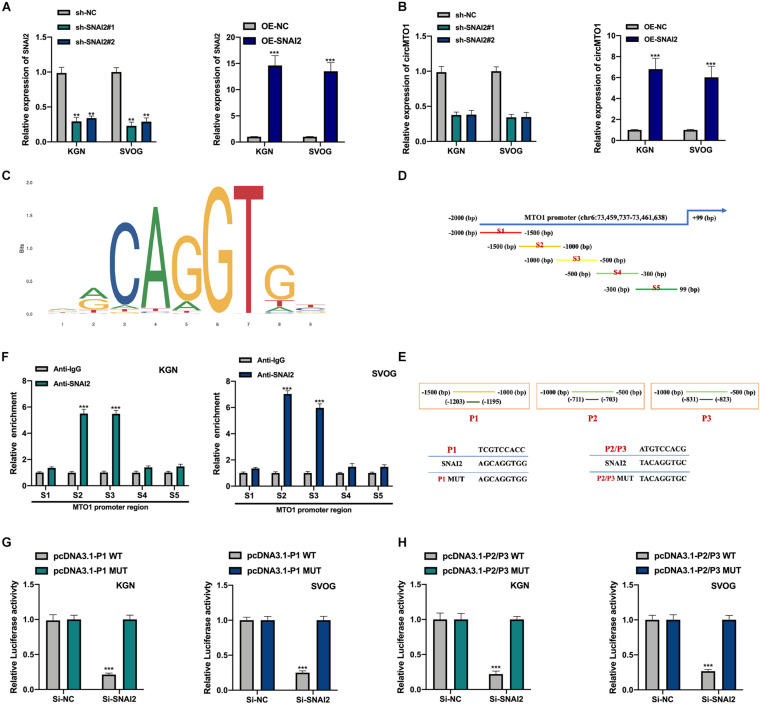
Induction of circMTO1 expression in Human granulosa-like tumor cells is induced by SNAI2. **(A)** Relative expression level of SNAI2 in SNAI2 knockdown or overexpressed Human granulosa-like tumor cells was measured using qRT-PCR. **(B)** Relative expression of circMTO1 in SNAI2 knockdown or overexpressed Human granulosa-like tumor cells was assessed using qRT-PCR. **(C)** The DNA motif of SNAI2 was showed. **(D)** The promoter of MTO1 was fragmented into S1-S5 regions. **(E)** The predicted binding sites between SNAI2 and MTO1 promoter. **(F)** The association between SNAI2 and S2/S3 region of MTO1 promoter was assessed by ChIP assays. **(G)** The association between SNAI2 and predicted P1 binding sites of MTO1 promoter were confirmed by luciferase reporter assays. **(H)** The association between SNAI2 and predicted P2/P3 binding sites of MTO1 promoter was proven by luciferase reporter assays. All experiments were conducted 3 times, **P< 0.01, ***P< 0.001.

We generated SNAI2 overexpression and knockdown cell models were generated (Figure 6A). SNAI2 positively regulated circMTO1 expression in human granulosa-like tumor cells (Figure 6B). Subsequently, the DNA motif of SNAI2 was obtained from the JASPAR dataset (Figure 6C). We separated the promoter sequence of MTO1 into five parts (S1–S5) (Figure 6D). The predicted binding sites between SNAI2 and MTO1 were located in S2 and S3 fragments (P1-P3) (Figure 6E). The association between SNAI2 and the S2 (P1) or S3 (P2/P3) region of the MTO1 promoter was verified using the ChIP assay (Figure 6F) and further confirmed by a luciferase reporter gene assay (Figures 6G,H) in human granulosa-like tumor cells. Therefore, our results revealed that the expression of circMTO1 in human granulosa-like tumor cells was facilitated by SNAI2.

## Discussion

Despite of the fact that PCOS is one of the most common endocrine diseases in women, and with an incidence of 6–12% (Yau et al., 2017), the etiology of PCOS is still poorly understood. On the one hand, the diagnosis and clinical intervention strategies for PCOS are inefficient and poorly improved. On the other hand, PCOS patients suffer varied presentations of PCOS clinical features including hyperandrogenism, oligomenorrhea, ovarian polycystic disorder, anovulation, infertility, and are facing multiple additive risks such as type II diabetes mellitus, metabolic diseases, hypertension and cardiovascular events (Azziz, 2018; Zhang J. et al., 2019). To improve the quality of life of PCOS patients, and reduce the economic burden of the world health care system, novel diagnosis or therapeutic targets for PCOS are urgently needed.

In this study, we verified that the expression and circRNA characterization of circMTO1 in human granulosa-like tumor cells. We also found that circMTO1 knockdown promoted cell proliferation and the inhibited the apoptotic cell rate in human granulosa-like tumor cells. Our results suggest that circMTO1 plays a role in the cellular processes of human granulosa-like tumor cells. Subsequently, the underlying molecular mechanisms are investigated. CircMTO1 sponged miR-320b in 293T cells and human granulosa-like tumor cells and negatively regulated miR-320b expression. We also identified MCL1 as a downstream target of miR-320b. Thus, CircMTO1 mediated human granulosa-like tumor cell progression through the miR-320b/MCL1 axis. Furthermore, we confirmed SNAI2 as an upstream transcription factor of circMTO1 in human granulosa-like tumor cells. We found a novel SNAI2/circMTO1/miR-320b/MCL1 axis in human granulosa-like tumor cell progression.

Evidence has revealed the biological functions of miR-320b in many diseases, such as osteosarcoma, psoriasis, osteoporosis, non-small cell lung cancer, and colorectal cancer (Wang et al., 2015; Lv et al., 2018; Wang Y. et al., 2018; Zhang L. et al., 2019; Zhang S. et al., 2019). Our findings revealed that miR-320b is a downstream factor of circMTO1 in human granulosa-like tumor cells. Its expression is suppressed by circMTO1. A previous study reported decreased miR-320b expression in ovarian cancer tissues, indicating that it might be a useful target for distant metastasis in ovarian carcinoma (Cha et al., 2017). Similarly, our results revealed that circMTO1 significantly downregulated miR-320b expression. However, the dysregulated expression of miR-320b in the ovary has different outcomes. The potential role and molecular mechanisms of miR-320b in ovarian disease require further study.

We also conducted biotinylated RNA pull-down and dual-luciferase gene assays, which confirmed that miR-320b directly targeted to MCL1. MCL1 has been found to be involved in the development of ovarian disorders in multiple ways, such as by deubiquitinating and interacting with miRNAs (Li C. et al., 2019; Wu et al., 2019). In this study, we transfected OE-NC, OE-circMTO1, OE-circMTO1 + si-NC, and OE-circMTO1 + si-MCL1 into human granulosa-like tumor cells, we found that the downregulation of MCL1 reversed the effects of circMTO1 on human granulosa-like tumor cell proliferation and apoptosis. Thus, circMTO1 exerts its functions in PCOS by regulating MCL1 expression.

Several studies have reported that transcriptional factors regulate circRNA expression in multiple cells (Wang R. et al., 2018; Li H. et al., 2019; Wang W. et al., 2020; Liu et al., 2021). Using the UCSC and JASPAR datasets of interest, we found that SNAI2 might be a promising transcription factor for the MTO1 promoter. We assessed and confirmed the association between SNAI2 and MTO1 promoters was assessed and confirmed using ChIP and luciferase reporter gene assays. Additionally, circMTO1 expression in human granulosa-like tumor cells was positively regulated by SNAI2.

## Conclusion

In conclusion, our findings suggest that circMTO1 could aggravate PCOS progression by upregulating MCL1 expression through interaction with miR-320b. Meanwhile, SNAI2 also induced the expression level of circMTO1 in human granulosa-like tumor cells. Our results provide new insights for human granulosa-like tumor cell research and may be a promising direction for PCOS intervention.

## Data Availability Statement

The original contributions presented in the study are included in the article/Supplementary Material, further inquiries can be directed to the corresponding author/s.

## Author Contributions

JD designed the experiments. JD and HC performed the experiments and analyzed the data. YH and LS collected and analyzed the experiments data. All authors read and approved the final manuscript.

## Conflict of Interest

The authors declare that the research was conducted in the absence of any commercial or financial relationships that could be construed as a potential conflict of interest.

## Publisher’s Note

All claims expressed in this article are solely those of the authors and do not necessarily represent those of their affiliated organizations, or those of the publisher, the editors and the reviewers. Any product that may be evaluated in this article, or claim that may be made by its manufacturer, is not guaranteed or endorsed by the publisher.

## Footnotes

1
http://miranda.org.uk/

2
http://starbase.sysu.edu.cn/

3
https://www.ncbi.nlm.nih.gov/

4
http://genome.ucsc.edu/

5
http://jaspar.genereg.net/

## Abbreviations

cicrRNA: circular RNA
PCOS: Polycystic ovary syndrome
qRT-PCR: quantitative reverse transcription polymerase chain reaction
FISH: Fluorescent *In Situ* Hybridization
RIP: RNA-binding protein immunoprecipitation assay
CCK-8: Cell Counting Kit-8
MCL1: MCL1 Apoptosis Regulator
GAPDH: Glyceraldehyde-3-Phosphate Dehydrogenase
circMTO1: circular RNA Mitochondrial TRNA Translation Optimization 1
SNAI2: Snail Family Transcriptional Repressor 2
siRNA: small interfering RNA.
