# Supplementary figures and images for: SNAI2-Induced CircMTO1 Promotes Cell Proliferation and Inhibits Apoptosis Through the miR-320b/MCL1 Axis in Human Granulosa-Like Tumor Cells
Source: Front Genet. 2021 Aug 3;12:689916. doi: 10.3389/fgene.2021.689916 (PMC8369758; doi:10.3389/fgene.2021.689916)

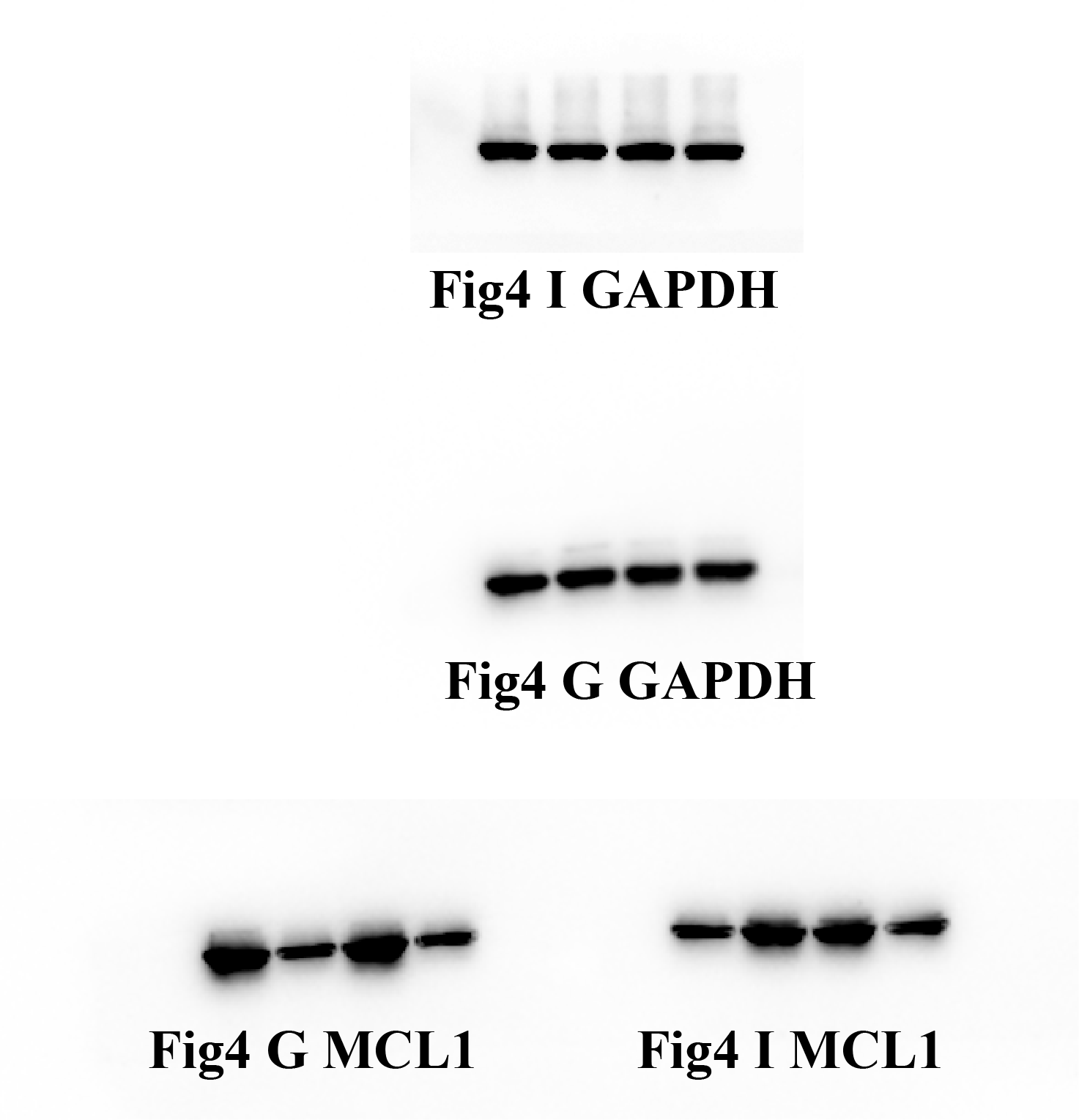

Supplement: Supplementary file 1 [file Image_1.TIF]
